# Effect of genetic testing for risk of type 2 diabetes mellitus on health behaviors and outcomes: study rationale, development and design

**DOI:** 10.1186/1472-6963-12-16

**Published:** 2012-01-18

**Authors:** Alex H Cho, Ley A Killeya-Jones, Julianne M O'Daniel, Kensaku Kawamoto, Patrick Gallagher, Susanne Haga, Joseph E Lucas, Gloria M Trujillo, Scott V Joy, Geoffrey S Ginsburg

**Affiliations:** 1 Center for Personalized Medicine, Duke University, Department of Medicine, Duke University School of Medicine, DUMC Box 90141, Durham, NC, USA, 27710; 2 Carolina Population Center, UNC-Chapel Hill, CB#8120 University Sq, 123 W Franklin Street, Room 304C, Chapel Hill, NC, USA, 27516; 3 CGC Illumina, Inc., San Diego, CA, USA, 92121-1975; 4 Department of Biomedical Informatics, University of Utah, 615 Arapeen Way, Suite 208 Salt; 5 Center for health Services Research in Primary Care, Durham VA Medical Center, 508 Fulton St. (152), Durham, NC, USA, 27705; 6 Center for Genomic Medicine, Institute for Genome Sciences & Policy, Department of Public Policy Studies Sanford School of Public Policy, Duke University, DUMC Box 90141, 304 Research Dr, North Bldg #228, Durham, NC, USA, 27708; 7 Institute for Genome Sciences & Policy, Duke University, 101 Science Dr, Rm 2121, Durham, NC, USA, 27708; 8 Department of Community and Family Medicine, Duke University School of Medicine, 2100 Erwin Rd, Marshall Pickens Building, DUMC Box 3886, Durham, NC, USA, 27710; 9 Center for Personalized Medicine, Duke University, Department of Medicine, Duke University School of Medicine, DUMC Box 3228, Durham, NC, USA, 27710; 10 Center for Personalized Medicine, Center for Genomic Medicine, Institute for Genome Sciences & Policy, Duke University, Departments of Medicine, Pathology, Duke University School of Medicine, 101 Science Dr, Rm 2111, DUMC Box 3382, Durham, NC, USA, 27708

**Keywords:** genetic information clinical utility, genetic testing, preventive health behavior, RCT protocol, risk perception, type 2 diabetes

## Abstract

**Abstract:**

**Background**

Type 2 diabetes is a prevalent chronic condition globally that results in extensive morbidity, decreased quality of life, and increased health services utilization. Lifestyle changes can prevent the development of diabetes, but require patient engagement. Genetic risk testing might represent a new tool to increase patients' motivation for lifestyle changes. Here we describe the rationale, development, and design of a randomized controlled trial (RCT) assessing the clinical and personal utility of incorporating type 2 diabetes genetic risk testing into comprehensive diabetes risk assessments performed in a primary care setting.

**Methods/Design**

Patients are recruited in the laboratory waiting areas of two primary care clinics and enrolled into one of three study arms. Those interested in genetic risk testing are randomized to receive *either *a standard risk assessment (SRA) for type 2 diabetes incorporating conventional risk factors plus upfront disclosure of the results of genetic risk testing ("SRA+G" arm), *or *the SRA alone ("SRA" arm). Participants not interested in genetic risk testing will not receive the test, but will receive SRA (forming a third, "no-test" arm). Risk counseling is provided by clinic staff (not study staff external to the clinic). Fasting plasma glucose, insulin levels, body mass index (BMI), and waist circumference are measured at baseline and 12 months, as are patients' self-reported behavioral and emotional responses to diabetes risk information. Primary outcomes are changes in insulin resistance and BMI after 12 months; secondary outcomes include changes in diet patterns, physical activity, waist circumference, and perceived risk of developing diabetes.

**Discussion**

The utility, feasibility, and efficacy of providing patients with genetic risk information for common chronic diseases in primary care remain unknown. The study described here will help to establish whether providing type 2 diabetes genetic risk information in a primary care setting can help improve patients' clinical outcomes, risk perceptions, and/or their engagement in healthy behavior change. In addition, study design features such as the use of existing clinic personnel for risk counseling could inform the future development and implementation of care models for the use of individual genetic risk information in primary care.

**Trial Registration:**

ClinicalTrials.gov: NCT00849563

## Background

The judicious use of genetic and genomic information has significant, if untested, potential to enhance the clinical care and prevention of chronic diseases - both indirectly, by contributing to our understanding of disease biology, as well as more directly, by providing additional information to influence providers' screening and treatment recommendations and patients' engagement and health behaviors [1].

Type 2 diabetes represents a highly relevant and important example of a genetically complex chronic disease. Over 20 million people currently suffer from diabetes in the US alone, and more than a million new cases are diagnosed each year [2]. High blood glucose is a leading cause of death around the world [3]. There is strong evidence that lifestyle changes can delay progression to diabetes, even in high-risk individuals, for at least a decade [4]. However, uptake of recommended behaviors appears disappointingly low, as evidenced by skyrocketing rates of obesity in the US and globally, driven by multiple factors [5-7]. Recent reviews of diabetes prevention and screening have thus articulated the need for more effective strategies for increasing patients' motivation and improving adherence to lifestyle changes which have been shown to reduce risk for type 2 diabetes [8,9].

To date, approximately 40 common DNA variants, or single nucleotide polymorphisms (SNPs), have been found to be associated with increased risk for type 2 diabetes [10,11]. One limitation to the broader application of such testing is that the magnitude of increased risk conferred by each variant is relatively small (although there is evidence that testing for these 40 variants can improve reclassification of diabetes risk for individuals under the age of 50 [11]). Thus, genetic risk testing for these variants may not yet offer significant predictive value for individual patients sufficient to alter providers' screening and treatment recommendations. Genetic risk information, however, could have clinical utility in other ways.

Clinical utility (defined as net benefit in improving health outcomes) of genetic testing could be demonstrated through increasing patient activation or positively influencing patient attitudes, beliefs, and health-related behaviors [12]. The relatively scarce research into clinical utility of genetic testing has produced mixed results [13,14]; however, some studies have found evidence that providing results of genetic tests for chronic diseases increases patients' preventive behavior [15,16]. A recent systematic review of the impact of genetic risk information on chronic adult diseases found some psychological benefits of including genetic information in treatment of chronic diseases, but concluded that many gaps in knowledge must be addressed before genetic science can be effectively translated into clinical practice [13].

In this paper, we describe the development, design, and methods of a randomized controlled trial (RCT) designed to assess the clinical utility of incorporating type 2 diabetes genetic risk testing into comprehensive diabetes risk assessments performed in primary care. The intervention tested in this RCT is integrated into a primary care setting and includes a theory-based tool to communicate risk of developing type 2 diabetes to patients. This study does not focus on the predictive utility of the genetic test *per se*; rather, it focuses on whether adding genetic test results to standard risk assessment affects patients' clinical outcomes, behaviors, and perceptions.

### Conceptual framework: Altering patient risk perceptions

A potential role for genetic risk information in disease prevention efforts is described by the Common Sense Model (CSM) of self-regulation of health and illness, adapted by Marteau and Weinman [17] to explain patient responses to health risk information. Health risk information (internal or environmental) informs the cognitive representation of and/or the emotions associated with a health threat, which in turn activates a coping plan, followed by an appraisal of the coping plan [18]. The appraisal then feeds back to update both the representation and coping plan in a continuous, dynamic fashion.

Genetic risk information could provide a distinct type of information to the cognitive representation of type 2 diabetes risk. For instance, the construction of beliefs about personal health risks may be derived in part from an individual's experience of the pattern of disease expression in their families [19]. However, patients may discount family history information because they feel different in crucial ways from affected relatives [20]. Genetic risk information that is specific to the individual cannot be discounted in the same way, and thus may carry added personal significance for patients. Genetic information might engender heightened perceived risk (i.e., anticipated harm if no action is taken), increased emotional response, and then formulation of a coping plan. Accordingly, our hypothesized mechanism of the effect of genetic risk information is that it increases perceived risk, which will in turn increase motivation to engage in coping by increasing preventive health behaviors. Genetic risk information may also serve as a more durable reminder of threat over time, and serve to reinforce the importance of the coping plan through the appraisal process [21].

## Methods/Design

This study is funded with support from The Duke Endowment (Charlotte, NC), approved by the Duke University Health System Institutional Review Board, and registered at ClinicalTrials.gov (NCT00849563).

### Overview

**Figure 1:**
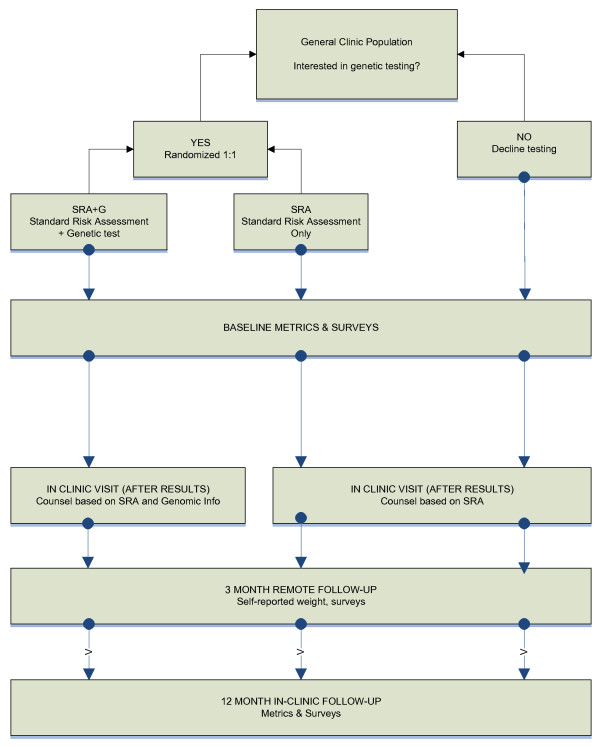
**Study flow**.

This RCT consists of three study arms (see Figure 1). Study coordinators tell prospective participants about the availability of genetic testing for diabetes risk, and ask them if they would be interested in undergoing such testing as part of a research study. After providing informed consent to participate and having eligibility confirmed, those interested in genetic testing are randomly assigned to undergo *either *a standard risk assessment (SRA) for type 2 diabetes plus upfront disclosure of the results of genetic risk testing (SRA+G), *or *the SRA alone (SRA). (The standard SRA incorporates the conventional risk factors of fasting glucose, body mass index (BMI), race/ethnicity, and age.) Patients not interested in genetic testing for diabetes risk are not randomized and receive only the SRA in a third study arm (No-Test).

Participants are also asked to complete four study encounters over 13 months: In-person visits at *baseline *and for *risk counseling *(approximately 4-6 weeks after baseline), a remote *3-month survey *(i.e., 3 months after risk counseling), and a *12-month *end-of-study visit. Height and weight (to calculate BMI), waist circumference, fasting plasma glucose and fasting insulin levels are measured at baseline and 12 months. Buccal swabs for DNA collection are obtained from all participants interested in genetic risk testing at the baseline visit. Surveys completed at each encounter will be used to track patients' behavioral and emotional responses to diabetes risk information over time, including perceptions of personal risk for type 2 diabetes. Primary outcomes are changes in insulin resistance and weight; secondary outcomes are changes in diet, physical activity, and waist circumference.

### Settings, eligibility, and recruitment

Patients are recruited in the clinical laboratory waiting areas of two primary care outpatient clinics - one an internal medicine practice, the other a family medicine practice -located in Durham County, North Carolina (NC), in the southeastern United States. These clinics serve a cross-section of Durham residents in terms of age, sex, race, and payor mix, and are affiliated with an academic health system. Durham County itself has a high prevalence of chronic disease and risk factors for disease. In 2007, 9% of all Durham County residents (14% of African-American residents) reported having a diagnosis of diabetes, 30% of residents were obese, and an additional 34% were overweight [22].

Study inclusion criteria are as follows: between the ages of 18 and 81 years; no self-reported history of diabetes; no self-reported history of prior genetic testing for diabetes risk; and not currently pregnant. To reduce the burden of participation, participants in the clinic who are already awaiting a fasting glucose draw or panel of tests that included glucose are invited to participate. Prospective participants are excluded if: they are taking or had taken medications normally used to treat diabetes; if baseline fasting glucose (tested at enrollment) ≥ 7 mmol/L (≥ 126 mg/dL), indicating possible undiagnosed diabetes; if they are not fasting and are unwilling to return for a fasting blood test; if baseline surveys are not completed; or if they cannot provide informed consent unassisted.

Participants will receive $20 for each of the three in-person study visits and are eligible for drawings for additional cash prizes for completing the *3-month survey *and *12-month visit*. In addition, those interested in genetic testing who are randomized to the SRA arm are given the option of receiving their diabetes genetic risk results after completing the *12-month visit *and concluding their participation in the study.

### Randomization

Randomization assignments are made when buccal swab samples are received from the two clinic sites at a central location, by study staff not installed at the clinical sites, and prior to sending the samples off for testing. This arrangement, in addition to obtaining buccal swabs from all participants interested in genetic risk testing, has the effect of blinding both participants *and *study staff at each clinic site to which arm participants have been randomized, until participants return for risk counseling.

### Genetic risk testing

Among the best-studied - and most predictive - SNPs associated with increased risk for developing type 2 diabetes, are two closely linked SNPs (rs7903146 and rs12255372) in the *TCF7L2 *gene; in both, the T allele is the higher-risk variant. More than 30 studies have replicated the finding that these two SNPs in *TCF7L2 *are associated with increased type 2 diabetes risk in White, Asian, and African American populations [23]. Participants in the Diabetes Prevention Program (DPP) study who were TT homozygous (i.e., had two higher-risk variants) at either of the two SNPs in the *TCF7L2 *gene had an increased risk of progression from impaired glucose tolerance to type 2 diabetes compared with participants who were CC and GG homozygous (with hazard ratios = 1.55 and 1.53, respectively) [24].

In the current study, one of these SNPs in the TCF7L2 gene, along with SNPs from three other genes (CDKN2A/2B, CDKAL1, and PPARG), reproducibly found to be associated with increased risk for type 2 diabetes, will be genotyped [10]. DNA is obtained via buccal swab from all participants who consent to genetic risk testing and is sent to a Clinical Laboratory Improvement Amendments (CLIA)-certified laboratory for testing (deCODE genetics, Chicago, IL). The conscious decision to use a commercially-available test from a CLIA-certified laboratory - rather than performing testing in a research laboratory - was made in order to maximize generalizability, and future translation, of any significant study findings.

### Family history

Self-reported family history of type 2 diabetes is assessed by asking respondents to report the number of first-degree (mother, father, brothers and sisters, children) and second-degree (maternal and paternal aunts, uncles, and grandparents) biological relatives with type 2 diabetes. Following the algorithm described in Hariri et al. [25], participants are classified into average, moderate or high familial risk for type 2 diabetes.

### Risk profiling

#### Diabetes risk profile

**Figure 2:**
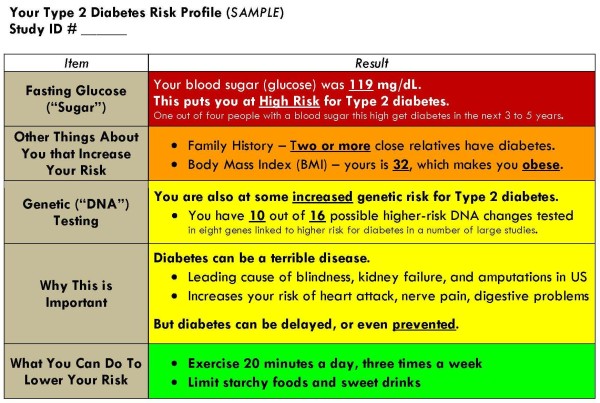
**Risk profile (used to communicate patient risk for developing type 2 diabetes)**.

At the time of their risk counseling visits, all participants receive a pictorial profile indicating their status with respect to individual risk factors for type 2 diabetes (see Figure 2). The profile used in this study was developed by a multi-disciplinary working committee, which included clinicians, genetic counselors, and a risk communication expert.

Age is included as a separate risk factor only for participants over 45 years of age. Race/ethnicity is included only when participants report Hispanic ethnicity, or African-American and/or Native American race, these being groups at elevated risk for type 2 diabetes.

In addition, each risk factor-containing box is color-coded to suggest different degrees of risk and aid in the communication of relative risks to the patient. For example, for the fasting glucose results, the background color would be red if the glucose test result is between 100 and 125 mg/dL, with the notation, "This means that you are 'prediabetic,' which puts you at High Risk for diabetes. You should probably see your doctor to follow up this test result. 1 out of 4 people with a blood sugar this high gets diabetes in the next 3 to 5 years." Conversely, the background color for this section would be yellow if the glucose test result is less than 100, with the notation, "This result falls in the normal range. What this means for your risk for diabetes depends on other risk factors, such as those below." BMI 30 and above (i.e., obese) would be colored red, between 25 and 30 (i.e., overweight) orange, and BMI less than 25 (i.e., normal) yellow. Age greater than 45 years of age and higher-risk race/ethnicity are color-coded orange.

Genetic risk appears on the profile only for participants randomized to receive upfront disclosure of genetic risk testing (i.e., those in the SRA+G arm). This is presented as the number of higher-risk alleles for each of the four SNPs included in the diabetes genetic risk test used in this study (i.e., "You have X out of 8 possible higher-risk DNA changes tested for in four genes linked to higher diabetes risk"). Participants homozygous for the higher-risk TCF7L2 (T) allele are given the additional information that overweight individuals with this genotype and prediabetes have approximately double the risk for progression to type 2 diabetes [24,26].

Participants receiving genetic risk results (SRA+G) are also given the official deCODE test report, which presents a combined genetic risk for type 2 diabetes as an odds ratio with an explanation of these results. Participants' primary healthcare providers are not given copies of either the risk profile or the deCODE test report, and participants are neither encouraged nor discouraged from sharing this information on their own.

### Web-based risk profile generator

Through iterative meetings led by a faculty informatician, specifications were developed to operationalize the profile as a web-based tool. A Web portal was then developed which collects these risk factors in a de-identified manner and generates a PDF risk profile based on these risk factors. The underlying technologies used in this Web portal were Java Server Pages for the Web development, Java for the software engineering, XML for the patient data representation, and XSL-FO in conjunction with the Apache Formatting Objects Processor for the conversion of the XML data into human-readable PDF risk profiles. The XSL-FO stylesheet was generated using commercial software (Altova StyleVision Enterprise Edition).

### Risk counseling

#### Training of embedded clinic staff

Embedded healthcare providers already involved in the clinical care of patients at each site - a clinical pharmacist (who is also a certified diabetes educator, or CDE) and a physician assistant at the family medicine clinic; and a nurse practitioner at the internal medicine clinic - have undergone a 2-hour training to ensure that risk information is consistently and accurately presented in easy-to-understand language and manner, with standardized content. The training session was developed and led by a certified genetic counselor, in consultation with a faculty researcher in risk communication, both of whom have extensive experience in genetic risk communication. Didactic, discussion, and role-playing activities focused on developing a knowledge base and strategies for communicating genetic and non-genetic risk information, as well as counseling regarding desirable lifestyle change [27,28].

#### Delivery of risk assessment results

Risk counseling appointments will be scheduled for each participant with one of the trained embedded healthcare provider(s) at that participant's regular clinic. These appointments will occur approximately 3-6 weeks after the baseline visit, sample collection, and return of genetic testing results (if any). To facilitate uniformity of counseling content, the risk profile described above will be used to guide the order of the visit.

#### Additional resources

Participants are provided with several additional resources to aid in the comprehension of diabetes risk information and implementation of diabetes prevention behaviors, including educational materials on diet, exercise, weight loss, and strategies for lowering risk for type 2 diabetes; a list of online resources compiled from standard diabetes education materials to facilitate active learning about type 2 diabetes, diabetes risk, and diabetes prevention; and the NHGRI publication *A Guide to Your Genome*, a 12-page booklet with information about genome science, genetic tests, and genome research. Participants are also provided with access to an online health portal, The PHD Network (Personal Health Development Network; http://www.activhealth.com). This health portal includes tools for assessing personal health risks for conditions including type 2 diabetes, as well as educational materials, health assessments, and behavioral goal-setting and tracking tools.

### Study measures: Outcomes

Primary clinical outcomes are changes in insulin resistance and weight over 12 months. Change in insulin resistance will be assessed by measuring fasting insulin and serum glucose levels at baseline and 12 months, and calculating the homeostasis model assessment of insulin resistance (HOMA-IR). The HOMA-IR is a widely-used measure of insulin resistance that is tightly correlated with the criterion standard of direct measurement by insulin clamp technique [29,30]. Change in weight will only be analyzed among overweight and obese patients (because healthy-weight patients will presumably not be counseled to lose weight). Weight at 12 months will control for baseline weight; specifically, the 12-month weight outcome variable will be residualized change scores (residuals captured from regressing 12-month weight onto baseline weight).

Secondary clinical outcome measures will be changes in diet, energy expenditure, and waist circumference. Change in diet will be measured using the 16-item dietary subscale from the National Health Interview Survey [31] and change in energy expenditure is measured using the World Health Organization's (WHO) Global Physical Activity Questionnaire [32]. Waist circumference will be measured at baseline and 12 months; the waist is defined as the midpoint between the highest point of the iliac crest and the lowest point of the costal margin at the midaxillary line [33]. All 3-month and 12-month outcome variables will be residualized change scores.

### Process Measures

Surveys will assess several process variables at several time points. *Belief in the role of genetics in type 2 diabetes risk *and *perceived personal risk for type 2 diabetes *are measured with newly designed items based on Common Sense principles. *Affective response to diabetes risk *are measured with the Consequences subscale of the Brief Illness Perception Questionnaire [34] and the Multidimensional Impact of Cancer Risk Assessment (MICRA) Questionnaire [35], both adapted to type 2 diabetes. *Perceived control over risk for type 2 diabetes *is measured with the personal control subscale of the Brief IPQ (adapted for type 2 diabetes).

### Analyses

Data will be analyzed according to intention-to-treat principles. Data will be checked for completeness and the characteristics and frequency of missing data will be described. Missing data values will be imputed as appropriate.

Our primary hypothesis is that patients in the SRA+G arm will have a greater reduction in insulin resistance and lose a greater percentage of their baseline weight after 12 months compared to patients in the SRA arm. We will test these hypotheses with linear multiple regression models with weight or insulin resistance at 12 months as the outcomes (testing a separate model for each outcome) and arm, clinic site, and baseline patient characteristics as explanatory and control variables. We will test our hypothesized mechanism with mediation analysis; specifically, hierarchical linear regression models will test whether effects of the intervention on insulin resistance and weight at 12 months is partially or fully accounted for by perceived risk at 3 months.

Our secondary hypotheses are that patients in the SRA+G group will report significantly healthier dietary changes and will have significantly greater average weekly energy expenditure than patients in the SRA group after 3 months. We will test this hypothesis with linear multiple regression models with behavioral variables at 3 months as the outcomes (testing a separate model for each variable) and arm, clinic site, and baseline patient characteristics as explanatory and control variables. We predict that this difference will narrow, but will still be significant at 12 months; we will test this hypothesis by fitting the same regression model with 12-month behavioral variables as outcomes. In addition, we hypothesize that overweight and obese patients in the SRA+G group, irrespective of diabetes genetic panel test result, will have a greater reduction in waist circumference. Residualized change scores (controlling for baseline values) will be used for 3- and 12-month outcome variables.

We will analyze the roles of process variables using moderation analyses in linear regression models. We predict (1) that patients who believe genetics plays a greater role in their risk of type 2 diabetes will be more likely to improve diet and exercise behavior at 3 months than those who believe genetics plays a lesser role in their diabetes risk; (2) that patients with higher perceived personal risk for Type 2 diabetes - regardless of actual test results, and regardless of group assignment - will be more likely to improve diet and exercise behavior at 3 months; (3) that patients with more positive affective responses (e.g., not anxious, guilty, or fearful) and more concern about their risk for type 2 diabetes - regardless of actual test results, and regardless of group assignment - will be more likely to improve diet and exercise behavior at 3 months; and (4) that patients with higher perceived control over their risk for type 2 diabetes will be more likely to maintain dietary and exercise behavior changes at 12 months.

### Power and sample size considerations

Power and sample size estimates are based on the primary study outcomes, percent weight change (from baseline) and change in HOMA-IR, both after 12 months. Based on two studies of low-intensity clinic-based weight loss interventions, we estimate that approximately 20% of patients in the SRA+G arm will lose 5% of their body weight over the course of a year, compared to approximately 10% of patients in the SRA arm [36,37]. Under these assumptions, the mean weight change for participants in the SRA+G arm is estimated to be around 1.7% of baseline body weight, with a standard deviation of approximately 3.9; and the mean weight change to be zero in the SRA arm. We further assume that changes in weight follow normal distributions with equal standard deviations for both groups. Because we are studying a low-intensity informational intervention, for purposes of sample size calculation we will aim for sufficient power to detect a difference in weight change that is 2/3 of the 1.7% difference estimated above. Thus, approximately 198 patients are needed in each group for a two-sided type I error rate of 0.05 and 80% power.

From a number of comparable studies, we conservatively estimate that participants in the SRA+G arm will be able to lower HOMA-IR by 0.5 over the course of a year through lifestyle changes; those in the SRA arm are again assumed to have a mean change of zero. Mean baseline HOMA-IR is estimated to be 2.5 with a standard deviation near 2.0 [38-41]. Using these estimates, 253 patients per group would be required to achieve a significance level of 0.05 with 80% power.

## Discussion

An important gap suggested by AHRQ-EPC [42] and USPSTF [43] reviews is the relative lack of effective strategies for increasing patients' motivation for and adherence to lifestyle changes known to reduce risk for type 2 diabetes. The study described herein examines a novel alternative strategy, in which patients are counseled about their germline genetic risk for type 2 diabetes as part of a comprehensive risk assessment. To our knowledge this is the first RCT to assess the *clinical utility *of disclosing the results of DNA testing for common variants associated with increased risk for type 2 diabetes. Study outcomes include patients' emotional and behavioral responses to this additional information, behavior changes (if any), and clinical endpoints. In addition, this study also addresses the *personal utility *of DNA testing. Our hypothesized mechanism of effect is that provision of type 2 diabetes genetic risk information during the risk counseling intervention will alter perceived risk, which will in turn increase motivation to engage in preventative health behaviors. Personal utility captures potential benefits of DNA testing that cannot necessarily be directly quantified in clinical outcomes [44].

Because this is one of the first trials to test for behavioral and clinical effects of communicating genetic test results, we carefully considered several novel and important issues during its development and design.

### Ethics of Communicating Genetic Risk Information

Adding genetic testing to standard risk assessment provides little improvement in the degree of absolute prediction of risk for developing type 2 diabetes. In designing the risk profile, we aimed to facilitate framing and evaluation of individual risk through suggestions of magnitude and meaning of results, and also to avoid falsely reassuring or overly alarming messages. By abstaining from presenting normative statements regarding the degree of genetic risk, we sought to keep participants from developing notions of fatalism or false security that might undermine motivation to change behavior [39].

### Strategy for Risk Communication

We aimed to simplify the presentation of risk information to be complete yet readily understood by patients. To this end, our risk profile (1) placed genetic risk information in context alongside other major risk factors for type 2 diabetes, (2) did not require a sophisticated understanding of either genetics or statistics, and (3) communicated personalized, evidence-based messages regarding individual risk which could then be further explained and amplified during the risk counseling visit (see Figure 2). This profile can readily be expanded to incorporate additional genetic risk markers for type 2 diabetes; and could even be adapted for use in other chronic diseases (e.g., coronary heart disease).

Combining different categories of risk information represented a particular challenge, especially given that patients' understanding and evaluation of their various risks are highly individualized. While the *magnitude *of risk may be a computable, objective quantity, the *meaning *of that risk for the individual is subjective and qualitative, and affected by many factors [45]. Genetic epidemiology has thus far almost exclusively focused on quantitative precision, and as a result, probably overemphasizes the significance of exactness when informing individual patients of their risks for a chronic disease. With this in mind, our risk profile was based on prospect theory, an established framework for understanding risk assessment and decision-making that has been previously applied to framing risk messages to promote healthy behavior [46,47]. Prospect theory posits two phases: editing (or framing) and evaluation; our risk profile was designed to address both.

The editing (or framing) stage is where the decision-maker's reference point is determined. We chose not to frame patients' risk information as a single quantity (i.e., "You have *x*% risk of getting diabetes in the next *y *years") for two reasons: First, there does not yet exist a comprehensive (prospective) risk prediction tool that combines and quantifies all of the known risk factors for type 2 diabetes, even excluding genomic risk. Second, individual differences in numeracy would likely have resulted in variation in understanding such a quantity [48]. Instead, we chose to simultaneously present patients with a number of distinct factors contributing to their overall risk for type 2 diabetes in relatively simple terms. Patients can evaluate each contributing risk factor in its own right, and each individual can arrive at an overall gestalt rather than having to interpret an abstract quantity.

Patients' valuing and weighting the overall risk for type 2 diabetes as a product of accepted, understood risk factors is described by prospect theory's evaluation phase. In our risk profile, different suggestive colors are used to highlight different degrees of risk for each factor. Simple quantitative reports and explanations are used to report blood sugar, number of relatives with type 2 diabetes, number of higher-risk alleles, and other risk factors (this "semi-quantitative" approach is based on the finding that individuals tend to gauge risk relative to other individuals or other risks [49]).

### Strengths of the Study

First, to increase the potential generalizability of the study to other primary care settings, we made the deliberate decision to use embedded clinicians already practicing at each clinic site to provide risk counseling, rather than inserting study personnel or referring study participants to outside genetic counselors. Second, to increase the potential for replication of any findings, we chose not to implement a supplementary behavioral intervention along with risk counseling. Although such a behavioral intervention might increase the effectiveness of risk counseling for increasing preventive behaviors, it also might have the effect of complicating the identification of unique effects of genetic testing. In addition, in many primary care settings, a discrete behavioral intervention may not be feasible due to cost and other resource constraints. Third, our risk counseling protocol is theory-based and relatively simple, maximizing the possibility that should the intervention prove successful, it might be more readily replicated in other settings with few additional resources required. In summary, although the main outcome measures are designed to test the effects of genetic risk information on clinical and behavioral outcomes related to prevention of a particular chronic disease (i.e., type 2 diabetes), results from this study could also inform the future development and implementation of care models for the use of individual genetic risk information in primary care more generally.

### Conclusion

The clinical and personal utility, feasibility, and efficacy of providing patients with genetic risk information for common chronic diseases in primary care remain largely unknown. The novel study described here aims to address these knowledge gaps, and the findings will contribute to the evidence base regarding the use of genetic risk testing to promote behaviors that reduce the risk of type 2 diabetes and other diseases. In addition, study design features such as employing existing clinic personnel for risk counseling and the creation of a comprehensive risk communication tool that includes both genetic and non-genetic factors, could be adapted for other settings and chronic conditions of interest, informing the future development and implementation of care models for the use of individual genetic risk information in primary care.

## Abbreviations

AHRQ-EPC: Agency for Healthcare Research and Quality-Evidence-based Practice Centers; BMI: body mass index; DNA: deoxyribonucleic acid; DPP: Diabetes Prevention Program; GWAS: genome-wide association study; HOMA-IR: HOmeostasis Model Assessment of Insulin Resistance; RCT: randomized controlled trial; SNP: single nucleotide polymorphism; SRA: standard risk assessment; SRA+G: standard risk assessment + upfront disclosure of genetic risk testing results (i.e., the SRA+G arm); USPSTF: United States Preventive Services Task Force.

## Competing interests

SVJ reports a relationship with deCode Genetics for support of testing, but no financial support. JO'D is employed by Illumina, Inc.

## Authors' contributions

AC, GSG and SVJ are responsible for the study design. SVJ and GMT facilitated the setup of recruitment in the two clinics where recruitment will occur. LKJ will be responsible for coordinating patient recruitment, data collection and data entry. AC, SH, JO'D, and SVJ developed the survey instruments used in data collection. AC, SVJ, LKJ, JO'D, and KK developed the risk profile algorithm and KK the web-based risk profile production tool. JO'D helped develop the risk counseling intervention and provider training. JEL was responsible for statistical analysis planning. GSG secured the funding that made this study possible and was the overall project principal investigator. LKJ wrote the first draft of the manuscript. PG contributed to statistical analysis planning and is the corresponding author. All authors read, revised, and approved the final version of the manuscript, for which AC is the guarantor.

## Pre-publication history

The pre-publication history for this paper can be accessed here:

http://www.biomedcentral.com/1472-6963/12/16/prepub
